# Cases in Practice

**Published:** 1860-12

**Authors:** F. K. Bailey

**Affiliations:** Joliet, Illinois


### CASES IN PRACTICE.

By F. K. BAILEY, M. D., JOLIET, ILLINOIS.

### *Hemorrhage from the Mouth Occuring Periodically.*

Case 1. Was called Wednesday, 17th, 1851, to visit C. K. æt. 27, large and robust; habits active. About the first of the month he began to feel soreness and stiffness in the region of the lower jaw of the left side, attended with pain in the back tooth. At first he paid but little attention to it, except the application of simple remedies to allay the uneasiness. Within a few days, however, there commenced a slight hemorrhage from the mouth, continuing with occasional interruptions, until I saw him, when it had become profuse.

I found him pale and feeble, but otherwise the functions were in a normal condition. The flow of blood had ceased before my arrival, and his mouth was stuffed with cotton, which I did not remove, for fear of breaking up the coagulum which had formed. I left him with directions to recall me if the blood should start again. The bleeding commenced again in a few hours, and continued till fainting resulted, when it again stopped, but I was not notified.

On Friday morning the 19th, I called and found there had been no hemorrhage since the fainting on the previous day. At this time I cleared his mouth of coagulum, and endeavored to find the orifice from which so much blood had escaped, but without success. I then left with special directions to call me should a recurrence take place. In the evening about eleven o'clock, I was summoned, and found the vital fluid running freely from his mouth. On examination found a strong pulsating point upon the ridge of the jaw immediately back of the tooth that had ached, and from this point the blood flowed per saltem at each beat of the heart. I extracted the tooth and the bleeding ceased immediately.

Saturday morning, 20th. Called and found the bleeding had not returned. Touched the point whence the blood had escaped, with a hot wire.

Sunday morning, 21st. Called and found there had been no return of hemorrhage. Left him with directions to be kept very quiet, and to have nourishing food.

Monday morning, 22d. Found the hemorrhage had returned at eleven the night previous, and continued through the night.

Suspecting periodicity, I prescribed sulphate quinine in three grain doses, to be given every two hours. Called at five in the evening, and found no return of the bleeding. Distinct pulsation at the affected spot. Applied the cautery, and directed sinapisms to be applied at the feet at nine o'clock, and that he should be kept warm and very quiet.

Tuesday morning, 23d. Called and found there had been no bleeding through the night. Patient refreshed by a good night's rest, the first he had enjoyed for a week. On examining the mouth, found the pulsation very distinct, the integuments upon the part affected being lifted up at each pulsation. Qunine in same dose every three hours through the day. At six o'clock in the evening found him comfortable, and directed sinapisms as before, and gave an anodyne. Applied the cautery.

Wednesday morning, 24th. No more hemorrhage, but the pulsation still continues.

From this time there was a rapid improvement, and with the use of tonic remedies, his health was soon restored. At the time of the first appearance of the hemorrhoge, there was no periodicity observed. It would occur at all times of the twenty-four hours, and continue but a short time, and in a slight degree.

For ten days the amount was so small that he kept about his business on the farm. It was not until after he had become considerably debilitated, that the hemorrhage occurred at stated periods, and until it returned the second time at eleven P. M. I had not thought of the case as one requiring antiperiodic treatment. The patient could in no way account for the first escape of blood, and there was nothing in the subsequent history of the case, to throw any light upon the matter.

I did not consider that the cautery had much, if any influence in stopping the bleeding, for the moisture of the mouth removed the eschar very soon after the first application. After getting the system under the influence of quinine, the escape of blood ceased, although there was determination to the spot, as indicated by the pulsation. I applied the cautery more at the solicitation of the family, than from any idea that it would prove effectual. Such an application was certainly a rational one, but in spite of it, the hemorrhage had come on after it was employed, and it was not until the patient was fully under the influence of quinine, that the bleeding was permanently arrested.

---

### *Hœmoptysis Occurring Periodically.*

Case 2.—Was called in the night, about the 15th October, 1848, to visit Mrs. D——, of middle age, and the mother of several children; constitution enfeebled by previous sickness, and of a scrofulous diathesis. The family lived five miles distant, and on my arrival I found that about midnight she had an attack of bleeding from the lungs. The hemorrhage had ceased, but she was extremely exhausted; the pulse very feeble and slow; countenance pale and haggard, and the extremities cold. She complained of pain in the left side about the lower edge of the mamma. There was some cough, with difficult expectoration. Had had cough and pain in the left side for some years, attended with muco-purulent expectoration. Menstruation had occurred one week previous to this attack. Dullness on percussion, with absence of respiratory murmur, indicated quite extensive disease of the left lung. Never had attack of bleeding before, or at least not to excite attention, as the blood had previously been mixed with the sputa.

By the employment of the proper means, she soon recovered from the condition of collapse in which I found her, and became warm. Left an infusion of serpentaria virginiana, to which was added muriate of ammonia, with a small amount of digitalis and epecac as an expectorant, with anodynes to be given as might be required. In three or four days she was able to sit up, and was soon about the house as usual.

In precisely four weeks from the time of her first attack, she had another, the symptoms being the same.

From this she gradually recovered. Anticipating a third attack after the same interval, I advised that she should be brought to the house of a friend near my own, that I might see her without delay in the event of another paroxysm. The attendants were also directed on the first approach of unfavorable symptoms, to place her feet in warm water, and to apply cloths wet in cold water to the chest and head.

As was anticipated, the symptoms of a third attack made their appearance after the expiration of four weeks as before. Instead of being in the night, as at the previous periods, she began to complain at four o'clock in the afternoon, thus anticipating about eight hours. I saw her in a few minutes, and found her complaining of a sense of fullness and pressure in the cheast and head, with coldness of the hands and feet. In other words the patient had a *chill.* No hemorrhage from the lungs occurred.

Her feet were in warm water when I called, and I immediately commenced pouring cold water upon the head, while cold compresses were placed upon the chest. Five grains sul. quinine with one grain piperine, were given at once, with a little brandy and water. In a short time there was complete reaction; but without much increased arterial action. Perspiration soon commenced, and she became comfortable. I then prescribed sul. quinine in doses of five grains, combined with an aromatic, to be given every four hours, for thirty-six hours.

From this time my patient began to improve, and by observing a similar precaution at the expiration of another four weeks, she escaped entirely anything like unpleasant feelings in the chest.

From that time to the present, I am not aware that she has had any hemorrhage from the lungs, but has continued feeble, and will undoubtedly sink sooner or later from pulmonary disease. I could not decide at the time whether the fact of recurrence once in four weeks was influenced by menstruation, or by the well known law, known to obtain in miasmatic diseases, of recurring at intervals of seven days, or an even number of sevens, or weeks, being four in this instance. Whatever may be true, however, the facts as they did occur are worthy of record.

---

*Chorea as an attendant upon Rheumatism.*

Case 1.—In June, 1858, I was called to visit a young lady æt. 17, and found her suffering from a severe attack of sub-acute rheumatism, involving the right foot and ankle. There was febrile excitement, frequent pulse, coated tongue, and great restlessness.

The remedies employed were opium and blue pill, to be followed with a laxative. Cloths wet with cold water were applied to the inflamed part. After the action of the laxative, a teaspoonful of the following mixture was directed to be given every four hours.

℞. Syr. Sarsa. Comp., ℥ ij.
Iodide Potassium, ʒ iss.

Alternated with the above were given two grains sul. quinine and six of Dover's powders. In four or five days the patient was able to sit up, and walk about the room. The local affection was nearly removed, and a rapid recovery anticipated. A slight relapse occurred, however, in about a week from the time of the first attack, when both feet became affected.

A continuance of the remedies first employed, but in increased doses, seemed to affect the local affection immediately; but there were indications of a cardiac complication. There was pain in the region of the heart, and severe palpitation. Full doses of opium soon allayed the pain, and to quiet the action of the heart. A mixture of equal quantities of fluid extract valerian and spts. nitr. dulc. was given freely.

After recovering partially from the last mentioned train of symptoms, it was noticed as she sat in a chair, that one foot would move convulsively. In a few hours the whole lower limb became so much affected as to render walking very difficult. The next day the muscles of the same side of the face became involved, and a severe case ef chorea was apprehended. Believing that these morbid appearances were the result of debility and general irritability of the system consequent upon the rheumatic attack, I prescribed the following mixture:

℞. Fluid ext. valerian, } ℥j. aa.

" " act. racemosa }

Syr. iodide ferri, ℥ss.

Sulphate quinine, ℈j.

To be taken in doses of a teaspoonful three times daily. The convulsive movements soon began to disappear, but continued to be apparent after she was able to walk about the city. As soon as her strength would permit, she went to Wisconsin, but continued to take the mixture in smaller doses for some time.

After an absence of two months she returned with her health restored. Not the slighest appearance of chorea has been seen up to the present time.

*Case* 2.—In July last, I was called to prescribe for a little girl aged eight years, and found her with some pain in the right foot and ankle, attended with considerable redness and swelling. In May or June she had rheumatism of the sterno-cleido-mastoideus muscle, rendering her neck inflexible for about three weeks. No other treatment was employed but such external applicatious as the parents made of their own suggestion. At the time I was called there was a strongly marked rheumatic diathesis, with an enfeebled constitution. The case was treated with iod. potassium and sul. quinine, in free doses, and no local applications except lotions of cold water if the heat was excessive. In about eight days the local affection which in the mean time had involved both limbs to the knees, suddenly disappeared, and she began to complain of severe pain in the pit of the stomach, with a palpitation so severe as to render it almost impossible to breathe. It was necessary to hold her in an upright position, for it was suffocation to be down. The pulse was 120 in a minute; the extremities cold; and the face and lips livid. The bellows murmur was very distinct, and endo-carditis seemed to be undoubtedly present.

I prescribed calomel and opium with sul. quinine in doses of one-third of a grain of the two former, with one grain of the latter every four hours, alternated with valerian and chlorate potassa. In about a month the violence of the symptoms was abated; the color returned to the face and lips; the palpitation became less, and she could lie down with partial ease.

Before the violence of these symptoms was lessened, however, unequivocal signs of chorea made their appearance. The right lower extremity was in constant motion, and it was extremely difficult for her to walk. The right arm also, was so much affected that she could not carry her hand to the mouth. The muscles of the feet were in constant motion, and the tongue so much involved, that she could scarcely articulate so as to be understood. This state of things continued for two or three weeks; but gradually as the little patient improved in strength, the affected muscles became completely under the control of the will. Subsequently, however, the cardiac symptoms became more severe, and failing slowly, she died on the 24th of September, without the least return of the choreic symptoms.

I find that Prof. Wood states that among other causes, chorea may result from translated rheumatism. In *Braithwaite* for Jan., 1854, Dr. Barcley, in speaking of a case of chorea, says: "She had had no rheumatism and the heart was healthy." Dr. Babington, in part 5th of *Braithwaite*, speaks of chorea arising metastasis of rheumatism to the theca of the spinal chord. Dr. Begbie, in part 15 of the same journal, speaks of chorea in connection with rheumatism. Also Dr. Chambers, mentions chorea with co-existent heart disease. Dr. Golding Bird mentions a case which supervened upon rheumatism. Dr.

Hughes, in Guy's Hospital reports for 1845, says: "Next to fright, rheumatism may be regarded among the most common causes of chorea." Dr. R. B. Todd, in *Med. Times and Gazette* for July, 1852, speaks of a case where symptoms of chorea supervened upon rheumatic fever.

During a practice of nearly a quarter of a century, I do not recollect having seen chorea supervening upon rheumatism, until case 1st came to my notice. In the cases mentioned, the choreic appearances did not appear until the heart became affected; and it would appear that such a phenomenon would not manifest itself except in metastasis. In both cases the chorea disappeared as the patient gained in strength, and in the second did not return, when the case assumed a fatal character. Other practitioners may have observed like cases; but if so, few have reported them, and the novelty, so far as my own observation is concerned, has induced me to send the above for publication.

---

